# Passing Events and Topics

**Published:** 1920-11-27

**Authors:** 


### Passing Events and Topics.

### Interchange of Foreign Degrees.

The Spanish Government has been requested by the Spanish Medical Association to abrogate the law permitting the interchange of Spanish and foreign medical degrees, owing to the serious competition of foreign doctors, particularly Germans and Austrians, who are entering Spain in large numbers.

### Hospital Pound Day.

The Pound Day, held on November 9 at the London Homœopathic Hospital, Great Ormond Street, W.C. 1, found a response in many friends of the hospital who were eager to bring some modest token of their sympathetic interest in the welfare of the sick patients, including a number of children from the local schools, who deposited their little offerings in the different receptacles with much enthusiasm. The gifts, which were of a varied nature, were received by the Countess of Donoughmore, and at the close of the day it was announced that the hospital had received 750 pounds in goods and £155 in money.

### Patients' Payments at the Middlesex.

With the exception of children under fourteen years of age, all out-patients at the Middlesex are to be asked to pay 6d. per attendance towards the cost of medicine, etc. In-patients will be expected to contribute in proportion to their means towards the cost of their maintenance while in the hospital or at the Convalescent Home at Clacton-on-Sea.

